# Supplementary material for: Combination of Silicate-Based Soil Conditioners with Plant Growth-Promoting Microorganisms to Improve Drought Stress Resilience in Potato
Source: Microorganisms. 2024 Oct 24;12(11):2128. doi: 10.3390/microorganisms12112128 (PMC11596784; doi:10.3390/microorganisms12112128)
Supplement: Supplementary file 1 [file microorganisms-12-02128-s001.zip › microorganisms-3227829-supplementary.pdf]

Supplementary data

Supplementary Table S1: Overview representing the experimental setup of the study

A) Overview Experiment I (vegatative growth, soil conditioners: Geohumus, Sanoplant)

Varients	Irrigation	Treatments
1	Well-watered	Control
2	Drought	Control
3		*Rhizophagus irregularis* MUCL41833 (AM)
4		*Rhizophagus irregularis* MUCL41833 + *Pseudomonas brassicacearum* 3Re2-7 (AM+3Re27)
5		*Rhizophagus irregularis* MUCL41833 + *Pseudomonas brassicacearum* 3Re2-7 + Soil conditioner Sanoplant (AM+3Re27+SP)
6		*Rhizophagus irregularis* MUCL41833 + *Pseudomonas brassicacearum* 3Re2-7 + Soil conditioner Geohumus (AM+3Re27+GH)

B) Overview Experiment II (generative growth, soil conditioner: Geohumus)

Variants	Irrigation	Treatments
1	Well-watered	PC Control
2	Drought	NC Control
3		Soil conditioner Geohumus (GH)
4		*Rhizophagus irregularis* MUCL41833 + *Pseudomonas brassicacearum* 3Re2-7 (AM+3Re27)
5		*Rhizophagus irregularis* MUCL41833 + *Pseudomonas brassicacearum* 3Re2-7 + Soil conditioner Geohumus (AM+3Re27+GH)

C) Overview Experiment III (generative growth, soil conditioner:Sanoplant)

Variants	Irrigation	Treatments
1	Well-watered	Control
2		*Rhizophagus irregularis* MUCL41833 + *Pseudomonas brassicacearum* 3Re2-7 (AM+3Re27)
3		*Rhizophagus irregularis* MUCL41833 + *Pseudomonas brassicacearum* 3Re2-7 + Soil conditioner Sanoplant (AM+3Re27+SP)
4	Drought	Control
5		*Rhizophagus irregularis* MUCL41833 + *Pseudomonas brassicacearum* 3Re2-7 + Soil conditioner Sanoplant (AM+3Re27+SP)

Supplementary Table S2: Cumulative fresh root and shoot biomass in drought stress experiments with potato with and without inoculation with *Rhizophagus irregularis* MUCL41833 (AM) + *Pseudomonas brassicacearum* 3Re2-7 and co-application of SC products (Geohumus®, Sanoplant® (relative changes % in brackets; * significant effects).

Shoot abd root biomass [g]	Control	Geohumus + AM +3Re27	Sanoplant + AM + 3Re27
	21.3	29.8 (+39.9%) *	29.9 (+40.4%*)
	27.4	35.2 (+28.5%)	31.9 (+16.4)
	53.6	109.3 (+103.9%) *	
	239.1	382.9 (+60.1%) *	
	187.1		212.9 (+13.8%)

Supplementary Table S3: Shoot K and Mn concentration and total content in potato leaves without application of microorganisms (Ctrl) and with application of *Rhizophagus irregularis* MUCL41833 (AM), *Pseudomonas brassicacearum* 3Re2-7 (3Re27), Sanoplant (SP) and Geohumus (GH). Different characters within the same column indicate significant differences between means (p≤0.05). t.

Treatment	K conc (mg g^{-1})	K content (mg $plant^{-1}$)	Mn conc (mg g^{-1})	K content (mg $plant^{-1}$)
Ctrl	46.6±2.7 b	92.4±6.1 b	0.041±0.001 d	0.081±0.005 d
AM+MP	45.3±2.1 b	110.9±5.3 ab	0.047±0.002 bcd	0.115±0.008 c
AM+3Re27	44.1±2.7 b	107.7±8.6 ab	0.043±0.003 d	0.105±0.008 cd
AM+3Re27+SP	46.9±1.2 ab	116.6±5.4 ab	0.045±0.002 cd	0.112±0.010 cd
AM+3Re27+GH	55.5±1.1 a	134.2±7.3 a	0.122±0.012 a	0.297±0.0367 a

Supplementary Table S4: Nutrient accumulation [mg $plant^{-1}$] in potato shoot tissue (dry matter) under well-watered and drought-stress conditions with and without application of microbial inoculants and the silicate-based soil conditioner Geohumus. AM: R. irregularis MUCL 41833 and 3Re27: P. brassicacearum 3Re2-7, GH: Geohumus. Different characters within the same column indicate significant differences between means (p≤0.05).

Treatment	P	K	N	Mg	Mn	Zn	Cu
well-watered	58.82±9.2 ab	1015±155 b	788±102 b	225±38 a	0.94±0.14 b	2.0±0.4 b	0.51±0.13 ab
drought stress	46.42±7.4 b	911±109 b	882±141 b	164±28 a	1.12±0.18 b	1.4±0.1 b	0.35±0.05 b
GH*	55.76±12.6 ab	1371±273 b	1197±222 ab	198±27 a	11.26±2.22 a	2.4±0.3 b	0.45±0.10 ab
AM+3Re*	53.16±7.8 b	1028±95 b	867±85 b	163±23 a	1.31±0.22 b	1.6±0.1 b	0.42±0.04 ab
AM+3Re+GH*	82.05±7.3 a	2036±485 a	1603±110 a	244±17 a	16.13±3.03 a	3.7±0.8 a	0.66±0.12 a

Supplementary Table S5: Nutrient accumulation [mg $plant^{-1}$] in potato shoot tissue (dry matter) under well-watered and drought-stress conditions with and without application of microbial inoculants and the silicate-based soil conditioner Sanoplant. AM: R. irregularis MUCL 41833 and 3Re27: P. brassicacearum 3Re2-7, SP: Sanoplant. Different characters within the same column indicate significant differences between means (p≤0.05).

A)	Treatments	Macronutrients (mg $plant^{-1}$)					Micronutrients (µg $plant^{-1}$)		
		P	K	N	Mg	Ca	Si	Mn	Zn
Well-watered	Control	46.9 b	759 bc	347 ab	54.6 ab	180 c	8.0a	247 c	815 abc
	AM+3Re27	53.3 a	884 a	345 ab	59.6 a	248 ab	8.9a	271 c	859 ab
	AM+3Re27+SP	40.9 b	662 bc	199 c	38.1 c	208 abc	9.1a	145 d	762 bc
Drought	Control	39.5 b	591 c	403 a	43.3 bc	193 bc	6.8a	449 a	665 c
	AM+3Re27+SP	36.2 b	629 bc	256 bc	32.8 c	165 c	10.4a	356 b	711 bc

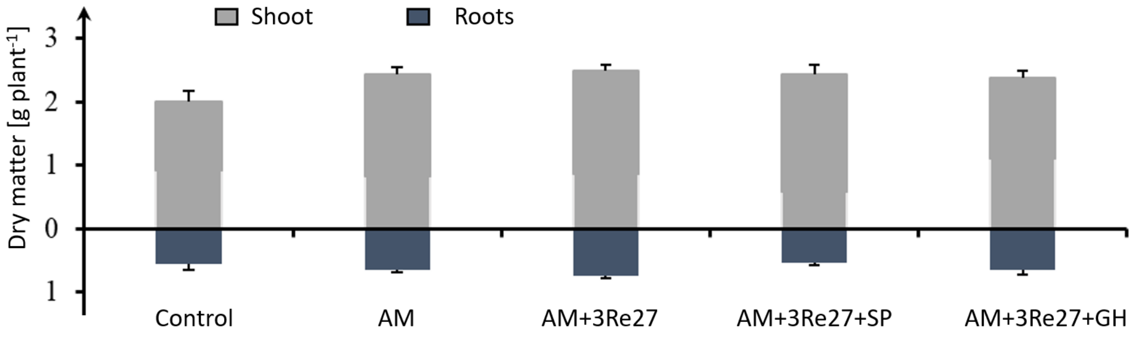

Supplementary Figure S1: Dry matter of potato plants after recovery from a 34 d drought stress period with and without application of microbial inoculants (*Rhizophagus irregularis* MUCL41833 =AM; *Pseudomonas brassicacearum* 3Re2-7 =3Re27) and silicate-based soil conditioners (Sanoplant = SP; Geohumus = GH). Means and SE of five replicates

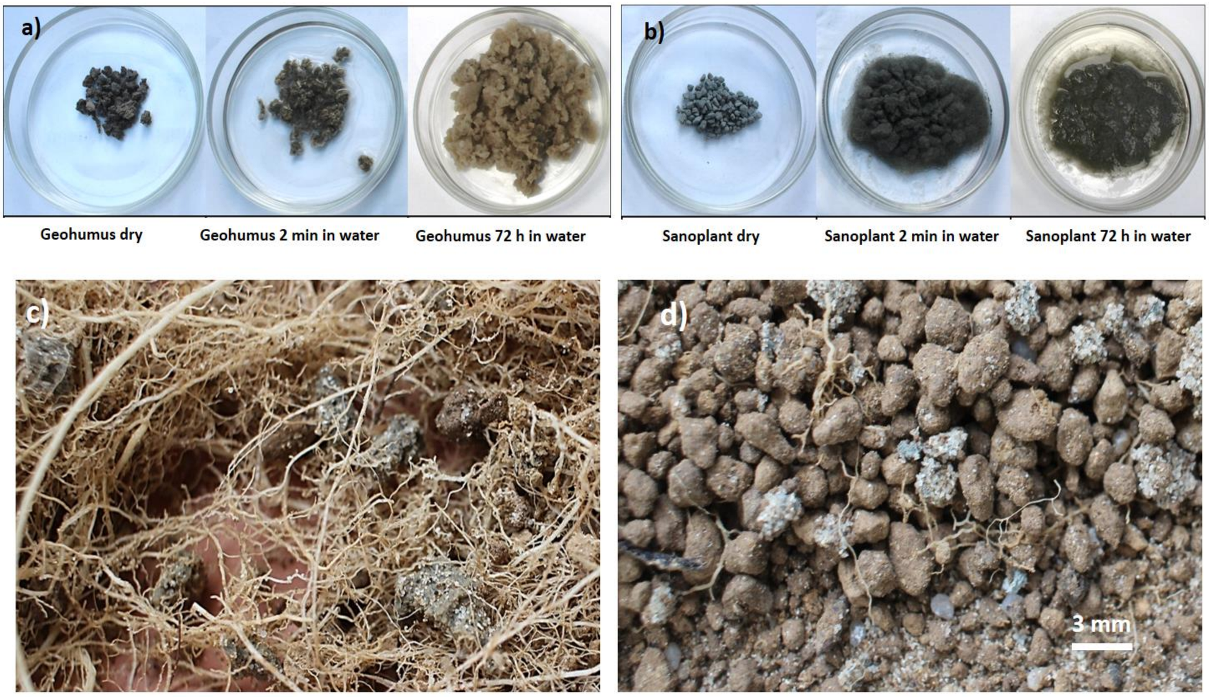

Supplementary Figure S2: Imbibition characteristics of Geohumus (a) and Sanoplant (b), Geohumus soil aggregates sticking to fine roots of potato plants (c) and soil particles (d).

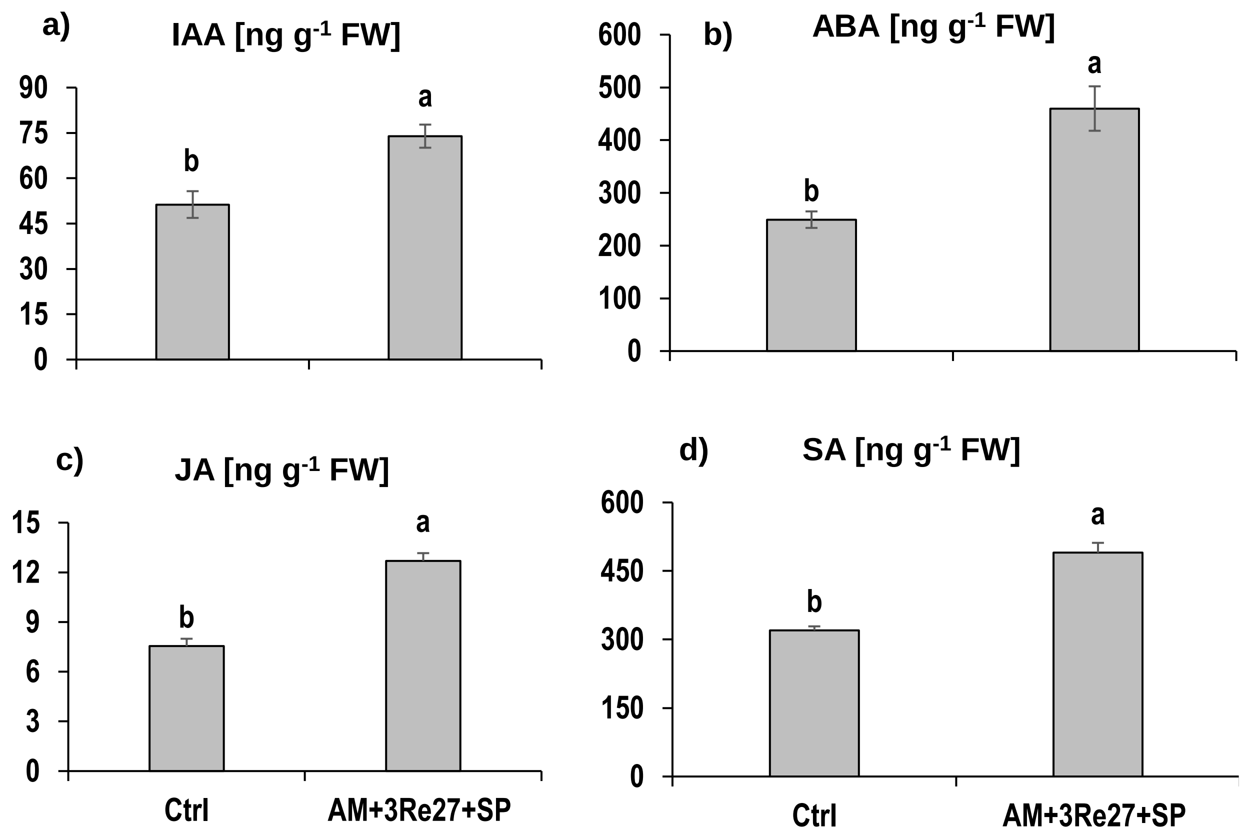

Supplementary Figure S3: Hormonal signatures in the root tissue IAA (a), ABA (b), JA (c) and SA(d) in drought affected potato plants supplied with microbial inoculants and soil conditioner Sanoplant [AM: *Rhizophagus irregularis* MUCL 41833 and 3Re: *Pseudomonas brassicacearum* 3Re2-7, SP Sanoplant.
